# An overview on the relationship between residential radon and lung cancer: what we know and future research

**DOI:** 10.1007/s12094-023-03308-0

**Published:** 2023-08-23

**Authors:** Alberto Ruano-Ravina, Lucia Martin-Gisbert, Karl Kelsey, Mónica Pérez-Ríos, Cristina Candal-Pedreira, Julia Rey-Brandariz, Leonor Varela-Lema

**Affiliations:** 1 Department of Preventive Medicine and Public Health, Facultade de Medicina, University of Santiago de Compostela, Rua San Francisco S/N, 15782 Santiago de Compostela, Spain; 2 Consortium for Biomedical Research in Epidemiology and Public Health (CIBER en Epidemiología y Salud Pública/CIBERESP), Madrid, Spain; 3 Health Research Institute of Santiago de Compostela (Instituto de Investigación Sanitaria de Santiago de Compostela-IDIS), Santiago de Compostela, Spain; 4 Cross-Disciplinary Research in Environmental Technologies (CRETUS), University of Santiago de Compostela, Santiago de Compostela, Spain; 5 Department of Epidemiology, Brown School of Public Health, Brown University, Providence, RI USA

**Keywords:** Radon, Lung cancer, Exposure, Molecular pathways

## Abstract

We aim to provide an overview of the research available on indoor radon and lung cancer, with a special focus on Spanish investigations. Early studies on underground miners established the link between radon and lung cancer, which was later confirmed for the general population by residential case–control studies. Spain contributed with extensive evidence, including 5 multicentric, hospital-based, case–control studies in the last 30 years, exploring diverse aspects, such as radon's effect on never-smokers, molecular pathways linking radon exposure to lung cancer risk, survival rates, mortality burden, and occupational exposure. There is a well-established causal association between radon with lung cancer. Despite pioneering research performed in our country by the Galician Radon Laboratory, particularly on driver genes, the evidence on the potential molecular pathways which makes radon a carcinogen is sparse. Also, relevant questions on the potential association of radon exposure with the induction of other diseases are still pending.

## Introduction

Radon is a colorless, tasteless, and odorless gas that comes from the disintegration of Uranium content in the rocks of the earth crust. In outdoor air, the concentration of radon gas is very low and poses no health risks. Nevertheless, in closed spaces (mainly cellars, ground and first floors of homes), it can reach concentrations which are dangerous to health. Since it is a gas, it can penetrate indoors through any hole, crack, or breach located on the basement or walls [1]. The main determinant of radon concentration indoors is the Uranium content in the underlying bedrock. A second factor that is determinant for radon exposure is how well homes and workplaces are isolated from the surrounding earth. If there is a good isolation, the radon concentration indoors can be low even when radon emanation from the earth crust in the area is high.

Radon was declared human carcinogen in 1988 by the International Agency for Research on Cancer (IARC) [2] and in 1987 by the US Environmental Protection Agency [3]. Radon gas is a source of ionizing radiation, because it disintegrates into other radioactive compounds releasing alpha particles which generate a significant health hazard. Alpha particles are highly energetic and a type of ionizing radiation with low capacity of penetration. Radon itself is not dangerous; its half-life is 3.8 days, but its descendants (sometimes called radon progeny) have a much shorter half-life and are solid [4]. Therefore, radon progeny can adhere to other small particles and remain in the lungs, exposing the lung to radioactive bombardment. In fact, the highest radiation dose received by the lungs comes from the decay of those short half-life descendants. If the indoor radon concentration is high, the cumulative dose of ionizing radiation received over time by inhabitants of this environment can be extremely elevated. As an example, the International Commission of Radiological Protection has established that a radon concentration at home of 300 Bq/m^3^ is equal to 14 mSv (milliSieverts) per year (more than a full chest CT) [5]; if such concentration occurs at work, the dose is equivalent to 4 mSv. Other equivalences establish that 148 Bq/m^3^ at home (which is the United States action level) is approximately the same dose as receiving 200 chest-X rays per year.

The US Environmental Protection Agency, the World Health Organization, and other international bodies agree that radon exposure is the main source of ionizing radiation exposure, noting that approximately 50% of all the ionizing radiation received during a lifetime is the result of radon exposure [6]. Nevertheless, in some countries, this is changing due to the rise in exposure to ionizing radiation from medical imaging tests. Nowadays, an average USA citizen receives more ionizing radiation from medical sources than from natural sources; however, this is not yet true of European citizens. However, taking into account the aging of the world population, the total dose of ionizing radiation per capita is expected to increase, given the higher use of such tests [7]. The dangers of ionizing radiation and its overuse are becoming a cause of concern for many organizations, including the American College of Radiology, who have promoted the campaign “Image Gently” to perform image tests only when needed [8].

In this paper, we aim to show an overview of the research performed on radon and lung cancer with a special focus on Spanish investigations, since this is the European country where most research has been executed in the last decades.

## Lung cancer epidemiology in short

Lung cancer is a public health problem with high incidence and mortality worldwide [9], including Spain. In fact, while in many developed countries, the incidence is decreasing due to tobacco control measures, in Spain, lung cancer incidence is decreasing in men but is strongly increasing in women. This increase has not yet reached its peak. Today, there are approximately 23,000 lung cancer deaths in Spain with an annual incidence of close to 30,000 cases. Most of lung cancer deaths occur in smokers and ex-smokers and there are important differences in the Spanish regions mainly due to their different patterns of tobacco use. Recent studies have shown that tobacco consumption causes approximately 60,000 annual deaths in Spain, and lung cancer is, by far, the disease which causes the largest fraction of tobacco-related mortality [10, 11].

## Radon exposure and lung cancer: available evidence

The first studies performed on radon exposure and lung cancer included miners of different uranium-containing ore in different countries in the 80s and 90s of the last century. Some of these studies comprised well-characterized cohorts of uranium miners, such as the Wismut Mining Company (Germany), Colorado Plateau (USA), or Czech Republic [12–14]. Most of these studies concluded that miners’ radon exposure increased consistently the risk of lung cancer, with no excess of risk for other tumors. These studies had a number of important limitations (mainly lack or poor adjustment for tobacco consumption, no women were included, and poor control for the simultaneous presence of other confounders and even carcinogens, i.e., dusts or gamma radiation exposure in the case of uranium miners). All these results were modeled by the BEIR VI report (Biologic Effect of Ionizing Radiations), a report by the National Research Council of the US, which concluded that there is a linear dose–response between radon exposure and lung cancer risk in general population [15]. This report was released in 1999, and this estimation was confirmed 6 years later with real data.

Following the miners’ studies, more research was performed, but in the general population, using a case–control study design. In such studies, generally, a radon device was placed at homes of lung cancer cases and controls, and most studies observed an association between radon concentration and lung cancer risk. Some seminal studies were performed in different countries, such as USA, Germany, France, etc. [16–18]. Spain also pioneered this research with a study performed in the Santiago de Compostela’s healthcare area, which included more than 400 cases and controls recruited between 1992 and 1994, 30 years ago. The results were published in 2002 and the resulting publication received the best paper award by the Spanish Society of Epidemiology [19]. The paper describes a significant association of radon exposure above 37 Bq/m^3^ with lung cancer with a submultiplicative interaction between radon exposure and tobacco consumption.

In 2005, the individual data of 13 European case–control studies were pooled in an European-wide research, leaded by Oxford University. The results were adjusted by sex, age, and tobacco consumption. A separate analysis was performed by histological type and the potential effect on tobacco and radon was thoroughly analyzed. The results were published in the British Medical Journal, and included more than 7000 lung cancer cases and 14,000 controls [20]. A more detailed and extended description of the results was published in the Scandinavian Journal of Work and Environmental Heath [21]. Perhaps, the most important result was the confirmation of a linear and statistically significant dose–response relationship between residential radon exposure and lung cancer risk, showing that for each 100 Bq/m^3^ of increase on radon concentration, lung cancer risk increased by a 16%. This is the most cited study on indoor radon and lung cancer and served as the bases for the World Health Organization (WHO) Handbook on indoor radon, released in 2009 [1]. The WHO report has been used later as the scientific ground to EU directive for the protection Against Ionizing Radiation, published in 2014 [22]. This is a good example of public health evidence-based policy. Also, in 2005, a similar study was published comprising the case–control studies performed in North America, but with a more limited sample size. These results were very similar to the European study [23].

## Indoor radon and lung cancer in Spain: what has been done up till now

Currently, Spain is the European Union (EU) country with most studies available on the relationship between radon and lung cancer, with studies performed in four Autonomous Communities which have included more than 4000 participants. An overall view of these studies appears in Table 1. At least 5 multicentric, hospital-based, case–control studies have been performed in the last 30 years, which have been published in high impact journals. Some of these studies have been awarded and all of them have been competitively funded. After the aforementioned research, carried out in 2022, a further study was performed between 2004 and 2008, including approximately 450 lung cancer cases and 550 controls in the healthcare areas of Santiago de Compostela and Ourense, observing again an association and a relevant interaction between radon exposure and tobacco consumption. The results were published in Cancer Epidemiology Biomarkers and Prevention, the official journal of the American Association of Cancer Research [24].

**Table 1:** Epidemiological studies on radon and health effects carried out in Spain

References	Title
Barros-Dios [19]	Radon in homes and risk of lung cancer: collaborative analysis of individual data from 13 European case–control studies
Ruano-Ravina [33]	Analysis of the relationship between p53 immunohistochemical expression and risk factors for lung cancer, with special emphasis on residential radon exposure
Llorca [34]	No evidence of a link between household radon concentrations and lung cancer in Cantabria, Spain
Ruano-Ravina [35]	Is there a specific mutation of p53 gene due to radon exposure? A systematic review
Barros-Dios [24]	Residential radon exposure, histologic types, and lung cancer risk. A case–control study in Galicia, Spain
Torres-Durán [25]	Lung cancer in never-smokers: a case–control study in a radon-prone area (Galicia, Spain)
Ruano-Ravina [36]	Genetic susceptibility, residential radon, and lung cancer in a radon-prone area
Ruano-Ravina [37]	Residential radon exposure and esophageal cancer. An ecological study from an area with high indoor radon concentration (Galicia, Spain)
Torres-Durán [27]	Residential radon and lung cancer characteristics in never-smokers
Ruano-Ravina [38]	Residential radon, EGFR mutations and ALK alterations in never-smoking lung cancer cases
Barbosa-Lorenzo [39]	Radon and stomach cancer
Lorenzo-González [40]	Residential radon in Galicia: a cross-sectional study in a radon-prone area
Ruano-Ravina [41]	Residential radon exposure and brain cancer: an ecological study in a radon-prone area (Galicia, Spain)
López-Abente [42]	Residential radon and cancer mortality in Galicia, Spain
Lorenzo-González [26]	Lung cancer and residential radon in never-smokers: A pooling study in the Northwest of Spain
Mezquita [43]	Indoor Radon in EGFR- and BRAF-Mutated and ALK-Rearranged Non-Small-Cell Lung Cancer Patients
Lorenzo-González [44]	Residential radon, genetic polymorphisms in DNA damage and repair-related
Ruano-Ravina [45]	Indoor radon in Spanish workplaces. A pilot study before the introduction of the European Directive 2013/59/Euratom
Casal-Mouriño [46]	Lung cancer survival in never-smokers and exposure to residential radon: Results of the LCRINS study
Gómez-Anca [47]	Radon Exposure and Neurodegenerative Disease
Conde-Sampayo [48]	Exposure to Residential Radon and COPD﻿: A Systematic Review
Rodríguez-Martínez [29]	Residential Radon and Small Cell Lung Cancer. Final Results of the Small Cell Study
Ruano-Ravina [49]	Lung cancer mortality attributable to residential radon exposure in Spain and its regions
Ruano-Ravina [50]	Indoor Radon Exposure and COPD, Synergic Association? A Multicentric, Hospital-Based Case–Control Study in a Radon-Prone Area
Mauriz-Barreiro [51]	Radon exposure and inflammatory bowel disease in a radon-prone area
Martin-Gisbert [52]	Lung cancer mortality attributable to residential radon: a systematic scoping review

In 2010, perhaps, one of the most interesting studies, which included exclusively never-smoking lung cancer cases and controls was undertaken. The final publication included more than 500 never-smoking lung cancer cases and more than 900 controls. It had a multicentric design and involved 11 hospitals from 4 different regions. This study comprises the second largest sample sized study published worldwide, analyzing radon and lung cancer exclusively in never-smokers. This study reported that radon was also associated with lung cancer in never-smokers, with a linear dose–response pattern and a significant risk above 200 Bq/m^3^. In accordance with the results of this study, if smoking is not present, a larger indoor radon concentration seems to be necessary to induce lung cancer [25–27] compared to what happens in former smokers. This study also observed that radon concentration appeared to be associated with an earlier age of lung cancer onset in never-smokers [27].

In 2013, a further study was initiated with the goal of assessing whether the association of radon exposure with lung cancer was modulated by genetic polymorphisms located in DNA-repair pathways, mainly BER and NER pathways. This study reported that certain genetic polymorphisms may modify the effect of radon exposure on the occurrence of lung and will be discussed later [28].

The last case–control study was exclusively focused on small cell lung cancer (SCLC). It had again a multicenter, case–control study design and involved hospitals from various regions. The study was conducted because of the lack of evidence on this specific and aggressive histological lung cancer type. This is the only study performed to date on radon and SCLC. It included 375 cases and 902 controls and again; it was found that radon increased the risk of SCLC with a dose–response relationship and an interaction with tobacco consumption [29]. Derived from this study, there were two more publications including exclusively SCLC cases who never smoked. Radon concentrations were quite high for some of these cases, suggesting that exposure may be a relevant cause of this disease [30, 31]. The results also served to shed light on the epidemiology on this neglected type of lung cancer [32].

With all this evidence available from robust research, it is clear that there is no need for more case–control studies to assess the relationship between radon and lung cancer. Nevertheless, there is still some uncertainty on how radon causes lung cancer, and different molecular pathways have been proposed. Areas in need of exploration include whether there exist any form of genetic susceptibility to lung cancer induced by radon exposure, the possibility of a specific hotspot in certain genes with a role in cell replication (e.g., TP53), and even some association with indoor radon and specific mutations in driver genes (e.g., EGFR) or genes involved in cell growth (e.g., ALK) and, which are a target for lung cancer treatment. In the next section, it will be discussed what is known on these potential molecular pathways.

## Available evidence on molecular pathways involving radon exposure and lung cancer risk

### Indoor radon and tumor suppressor genes

There is a body of literature suggesting that indoor radon might be associated with some hotspot mutations in TP53 gene, (reviewed in [35]). This hotspot has been observed with tobacco consumption, but there are doubts on the effect of radon exposure. TP53 is the most commonly mutated gene in many human cancers, and lung cancer is not an exception [53]. The polyaromatic hydrocarbon (PAH) benzo[a]pyrene, which is metabolically activated to benzo[a]pyrene-diol-epoxide (BPDE), preferentially binds the N2 position of guanine at TP53 codons 157, 248, and 273 in normal human bronchial epithelial cells. These bulky DNA adducts generate a smoking signature described as G:C > T:A transversion mutations, which is sometimes characteristic in smokers, though is dependent on DNA-repair mechanisms. Other occupational exposures have been suggested to be associated with TP53 mutations already in 2003, but progress in this area has been scarce [54].

Previous studies have also associated radon exposure at high concentrations in miners with a potential hotspot at codon 249, although this has not been found consistently. The first study, performed in 1992, sequenced the TP53 gene from a small sample of lung cancer cases. None of the mutations were G:C to T:A transversions in the coding strand of the TP53 gene, which are the most frequent base substitutions associated with tobacco smoking, and the authors concluded that the observed differences from the usual lung cancer mutational spectrum may reflect the genotoxic effects of radon [55]. A following study performed by Taylor and colleagues in a small sample of uranium miners suggested that there was the same signature in 31% of squamous and large cell lung cancers [56]. Nevertheless, this was refuted by other authors, because genetic damage from ionizing radiation is expected to induce primarily DNA strand breaks, and to be more random in its DNA damaging and mutagenic effects [57]. This mutation was not found in studies from miners with lung cancer belonging to the Wismut Mining Company in Saxony, Germany. Of note, both studies included approximately 50 participants. A subsequent research observed that, analyzing 23 lung adenocarcinomas from the same cohort of highly exposed uranium miners, 39% of these tumors contained one or more mutations within hotspots in the K-RAS gene [58].

Other studies have assessed whether indoor radon exposure in residential settings was associated with the presence of mutations in TP53 or other genes. The study by Yngvesson et al. included 83 never-smoking lung cancer cases and 250 ever smoking lung cancer cases. They found more TP53 mutations in ever-smokers compared to never-smokers and a somewhat higher frequency of mutations in those with higher radon exposures, particularly in never-smokers but without a clear mutation pattern [59]. Later, a study was performed in Galicia analyzed using immunohistochemistry and sequencing TP53 mutation patterns with indoor radon exposure. It included 72 patients and observed no association between a more intense staining and radon exposure. There was also no evidence of mutations related with radon [33]. A systematic review also performed by Spanish authors regarding the association between TP53 mutations and radon exposure that included eight studies with a total of 578 individuals. Twenty-six percent of radon-exposed miners' tumors had a mutation in gene TP53, versus 24% in the population exposed to residential radon. A predominance of the AGG (ARG) ⟶ ATG (MET) (arginine-to-methionine) mutation in miners was observed. Nevertheless, the authors concluded that the existence of such association between TP53 mutations and radon exposure was unclear [35]. Though the evidence is still scarce, more recent studies have been published but again with inconclusive findings [60, 61]. There is only one study performed examining K-RAS mutation and radon exposure, observing a possible association between radon and downregulation of this gene [62]; however, this work was performed in animal model.

### Indoor radon and driver genes

Regarding Epidermal Growth Factor Receptor (EGFR) and radon exposure, there are few studies available, though the first one was published more than 10 years ago. Taga et al. analyzed 70 adenocarcinomas in never and former smoking women observing that radon exposure was not consistently associated with EGFR mutations [63]. Of note, in this study, some differences were observed regarding radon quartiles. A study published in 2016 by Ruano-Ravina et al. included 323 never-smoking lung cancer patients [38]. This report found that 42% of participants were EGFR positive. The most frequent EGFR alterations were exon 19 deletions and exon 21 (L858R) single-point substitution mutations. Residential radon levels were twofold higher in patients with exon 19 deletions compared with patients with exon 21 (L858R) single-point substitution mutations (216 versus 118 Bq/m^3^), a result close to statistical significance, but there were no differences in residential radon levels by EGFR mutation status. These results suggest that radon exposure might be associated with specific EGFR mutations. Finally, a smaller study including 48 patients and performed in Madrid (with detectors assessed at the Galician Radon Laboratory) did not find any association of indoor radon and EGFR mutations. It is important to mention that indoor radon concentrations in this research were low and had a limited sample size [43].

### ALK and indoor radon exposure

To our knowledge, only two studies have investigated whether indoor residential radon might be related with the well-known ALK gene translocation. The first and largest sample size study was published in 2016, including 80 never-smoking lung cancer patients who had ALK translocation assessed. Twelve of them were positive, and they had twofold higher measured residential radon levels compared with ALK-negative patients (290 versus 164 Bq/m^3^, respectively) [38]. A smaller study which analyzed 10 patients for ALK translocations did not find any association with radon exposure [43]. Overall, these results suggest that radon may have a role in the molecular signature of lung cancer in never-smokers, although more studies with larger sample sizes are needed to support this hypothesis.

## Indoor radon and genetic susceptibility to lung cancer

Lung cancer is a multifactorial disease. It has many different risk factors, besides tobacco consumption and indoor radon exposure. The question why only a percentage of smokers develop lung cancer (estimated approximately in a 15%) remains, and it is thought that this is due to numerous factors, including how the human body metabolizes different carcinogens, and how it repairs DNA aggressions in the form of DNA adducts and strand breaks. The combination of different polymorphisms in multiple susceptibility genes results in variability on lung cancer risk in individuals smoking the same number of cigarettes per day (or perhaps exposed to the same radon concentration). It is also well known that lung cancer is a disease where no high-penetrance genes have been discovered (with the exception of certain syndromes or diseases increasing cancer risk such as Li–Fraumeni or Retinoblastoma) [64]. This means that a multigenic approach strategy is necessary to ascertain how environmental carcinogenesis is mediated through different genetic polymorphisms.

The literature is still scarce regarding radon exposure and susceptibility genes, though scientific evidence is being accumulated on the potential role of certain genes. It is still not known if these genes can increase lung cancer risk due to radon exposure just because they increase the risk in ever-smokers or if they, per se, increase radon-related lung cancer risk. The first candidate genes to be proposed to have a role in radon-related carcinogenesis were phase II genes *GSTM1* and *GSTT1* (Glutathion-S-Transferase M1 and T1); these genes both have deletion variants of appreciable prevalence in different populations. They are known to make carcinogen metabolic intermediates coming from tobacco products more water soluble and therefore easily eliminated through urine. The first study was published by Bonner et al. in 2006 and it was observed that radon concentrations higher than 121 Bq/m^3^ were associated with a more than threefold risk (OR = 3.41; 95% CI 1.10, 10.61) for *GSTM1* null homozygotes compared to *GSTM1* carriers [65]. This study had a case-only design including 270 lung cancer cases, and suggested that radon effect might be modulated by *GSTM1*. The second study was published by Ruano-Ravina et al. in 2014 in the Journal of Thoracic Oncology. It included 792 participants and had a case–control design [36]. Carriers of *GSTM1* variant and residential radon concentrations higher than 148 Bq/m^3^ had an OR of 1.48 (95% CI 0.73–3.00), whereas those with *GSTM1* deleted had an OR of 2.64 (95% CI 1.18–5.91) when compared with participants with *GSTM1* present and radon concentrations below 50 Bq/m^3^. Similar results were observed for GSTT1 deletion. These results suggest that those individuals null for *GSTM1* and *GSTT1* might be more susceptible to develop lung cancer. Unfortunately, this study included ever and never-smokers, so the effect in never-smokers could not be analyzed separately due to a low sample size of this subpopulation. A 2016 study performed by Sinitsky et al. in Russian children in a high radon area assessed the effect of the following genes: *ADPRT* (rs 1136410), *hOGG1* (rs 1052133), *NBS1* (rs 1805794), *XRCC1* (rs 25487), *XpC* (rs 2228001), *XpD* (rs 13181), and *XpG* (rs 17655). They observed a higher frequency of binucleated lymphocytes with micronuclei (MN) in carriers of the His/His genotype of the *XpG* gene Asp1104His polymorphism in comparison to heterozygous and homozygous carriers of the Asp allele. This was a descriptive study and no direct measures of indoor radon were obtained, though the authors suggested some association between radon and these alterations [66].

In 2019, a Spanish study, this time performed exclusively in never-smokers, analyzed different genetic polymorphisms and radon exposure. It included *GSTT1*, *GSTM1*, *XRCC1* (rs25487), *ERCC1* (rs11615, rs3212986), *ERCC2* (rs13181), *XRCC3* (rs861539), *OGG1* (rs1052133), and Alpha-1-Antitrypsin mutations (*AAT*) [44]. This study was multicentric, multi-region and included 660 participants (322 cases). The results were very interesting, because suggestion of association was again observed for *GSTM1* and other polymorphisms. The Odds Ratio (OR) for deleted *GSTM1* patients was 3.46 (95% CI 1.52–7.89) at residential radon exposures above 200 Bq/m^3^. The *ERCC1* rs3212986 polymorphism was the most associated with the risk of developing lung cancer, both for low and high radon exposures. The *ERCC1* rs321986 GT and TT genotypes (at radon concentrations > 200 Bq/m^3^), with ORs being, respectively, 2.40 (95% CI 1.29–4.45) and 4.45 (95% CI 1.26–15.7). This study included not only genes involved in the metabolization of carcinogenic compounds but also genes involved in DNA repair from external aggressions, which is the case of *ERCC1*, *ERCC2*, and *ERCC3* and *XRCC3*. It is important to mention that there is a relevant advantage from including exclusively never-smokers, because these results are not blurred by the presence of carcinogenic compounds coming from tobacco consumption. Again, these results suggest that radon effect may be mediated by individual differences.

Finally, a paper published in 2022, using a multicentre, hospital-based, case–control study included 826 cases and 1201 controls was focused on analyzing DNA-repair mechanisms related to radon exposure and analyzed 24 single-nucleotide polymorphisms (SNP) in fifteen genes and one SNP in a non-protein-coding RNA (ncRNA) region of chromosome 18 involved in DNA repair. The genes and rs (rs-reference for Single-Nucleotide Polymorphism) analyzed involved in DNA repair were: *ERCC1* (rs11615, rs3212986), *ERCC2* (rs13181, rs1799793), *ERCC3* (rs3738948, rs4150459), *ERCC5* (rs1047768, rs2094258), *OGG1* (rs1052133, rs2072668, rs2472037, rs125701), *APEX1* (rs1130409, rs3136817), *XRCC1* (rs25487), *XRCC3* (rs861539), *MUTYH* (rs3219489), *NBN* (rs1805794), *RRM1* (rs12806698), *XPC* (rs2228001), *KLH4* (rs5922437), and *FATS* (rs11245007). In addition, we included in this study the single-nucleotide variation in intronic region rs1452584, located in chromosome 18, q21.33, which is in an ncRNA gene, these regions might have regulatory functions. Besides showing an effect in different smoking categories, some genes were associated with radon exposure (using as a cutpoint 200Bq/m^3^). Some of these associations showed a statistically significant association. This study is the most extensive study performed to date regarding both the number of genes analyzed and sample size included [28].

## Indoor radon and lung cancer survival

To our knowledge, only one study has analyzed whether indoor radon exposure is associated with lung cancer survival. This study was performed exclusively in never-smokers and showed that 3- and 5-year survival was significantly associated with indoor radon exposure. The study included 369 never-smoking lung cancer cases. Those participants who had radon concentrations higher than 300 Bq/m^3^ had 1.5 higher risk of death, after adjusting for stage at diagnosis, age, sex, and other treatments received (surgery, chemotherapy or radiotherapy). More research is needed to confirm these results which add a further research line to the potential effects of radon on lung cancer [46].

## Radon distribution and impact on attributable mortality: the specific example of Spain

Radon distribution is highly variable worldwide. Some regions have been well characterized as radon-prone areas in Europe and in other parts of the world. Some examples are the Rocky Mountains, the Northeast and Appalachian regions in the US, Central France, Southern Germany, and other different countries [67]. We can say that practically all medium-size countries have a high radon area. This different radon distribution has also an impact on radon attributable lung cancer deaths, as we have published recently [52].

**Fig. 1:**
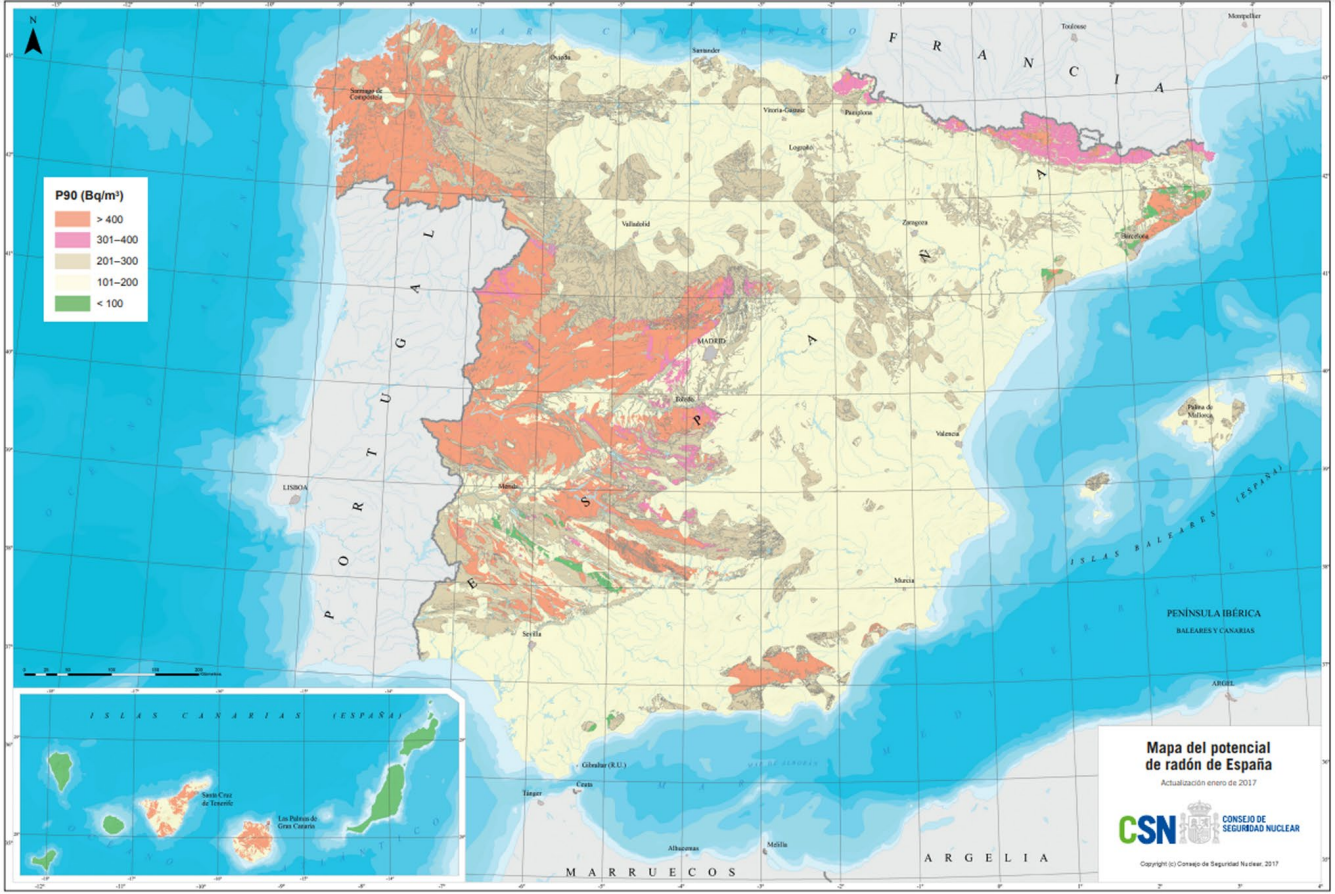
National radon potential map of Spain by the nuclear safety council (CSN) [68]. The radon potential is presented here as percentile 90 (P90) value for radon concentration. For instance, in an area with a P90 of 400 Bq/m^3^, 90% of homes measured are below or equal to 400 Bq/m^3^, or in other words, 10% of homes exceed 400 Bq/m^3^

Radon distribution is heterogeneous in different regions. In Spain, according to the Nuclear Safety Council, 70% of the Galician territory is affected by high radon concentrations, followed by Extremadura (47%) and Madrid (36%) [68]. Figure 1 shows the radon distribution in Spain, comprising approximately 12,000 indoor radon measurements.

At the national level, a recent report commissioned by the Ministry of Health to the Galician Radon Laboratory estimated attributable lung cancer deaths due to radon exposure overall and by region [49, 69]. Of all lung cancer deaths in Spain, it is estimated that 3.8% are attributable to indoor radon exposure. By Autonomous Communities, this figure would be 7% in Galicia, followed by Extremadura (6.9%) (see Table 2). 838 annual lung cancer deaths could be attributable to radon exposure throughout the country. This study has been the first performed taking into account the population living in upper dwellings. If we consider only population living at the ground level (ground or first floors), these figures would be twofold, reaching 13% of lung cancer attributable mortality in Galicia and Extremadura. This is an important detail, since we can assume that in high radon emitting regions that are in rural areas, people are more likely to live very close to the ground floor, implying that radon exposure may have a significant impact on lung cancer mortality in these regions.

**Table 2:** Lung cancer mortality attributed to radon (as Population Attributable Fraction—PAF and attributable mortality—AM) for each Autonomous Community in Spain, for males and females in 2017 [49]

Autonomous communities	PAF (%)	Males AM	Females AM
Andalusia	3.8	110	24
Aragon	2.9	18	4
Asturias, principality of	2.8	15	5
Balearic Islands	3.7	14	5
Canary Islands	5.5	36	13
Cantabria	3.4	9	3
Castile and Leon	4.7	49	13
Castile-La Mancha	5.2	44	8
Catalonia	3.6	92	28
Valencian Community	2.7	51	14
Extremadura	6.9	37	6
Galicia	7.0	86	24
Madrid, Community of	2.3	44	16
Murcian, Region of	3.7	17	4
Navarre, Foral Community of	2.9	7	2
Basque Country	1.8	17	6
Rioja, La	2.8	3	1

Lung cancer mortality attributed to radon exposure is presented here as: percentage of total lung cancer deaths, this is the Population Attributable Fraction (PAF%); as number of lung cancer deaths attributed to radon, this is the Attributable Mortality (AM)

The best-characterized Spanish region regarding indoor radon is Galicia, which has also proven to be an area of quite elevated radon, as commented before. Galicia has its own indoor radon map, which was started in 2001 by the University of Santiago de Compostela (Galician Radon Laboratory) [40]. Currently, the Galician Radon Map has 6080 indoor radon measurements in all Galician municipalities. The map has evolved obtaining measurements in each census tract. Close to a third of Galician municipalities have at least two radon measurements in each one and we consider them completed. Table 3 shows the radon distribution by province. The provinces with the highest indoor radon concentrations are Ourense and Pontevedra, with approximately 20% of all the measured dwellings having more than 300 Bq/m^3^, which is both the EU and Spanish action level.

**Table 3:** Radon in the 4 Galician Provinces: percentage of homes exceeding 300 Bq/m^3^. *Source*: Data from Galician Radon Laboratory (https://radon.gal/)

Galician provinces	*N*	% exceeding 300 Bq/m^3^ (%)
A Coruña	2629	16
Lugo	651	11
Ourense	801	23
Pontevedra	1999	21
Total Galicia	6080	18

## Is radon a problem at work?

There are different studies analyzing radon exposure in the workplace. The first studies linking radon with lung cancer were performed in miners [70–72]. Subsequently, more research pointed to other occupations with a high radon exposure apart from miners. These include underground occupations and other specific workplaces (i.e., parking lots, underground galleries, those associated with hot springs, and many others) [73–75]. The overall evidence suggests that some workplaces may have substantial potential for quite high radon exposure, particularly those located in ground floors and in radon-prone areas. A recent study conducted in more than 3000 Spanish workplaces has found that close to 20% of workers in radon-prone areas are exposed to radon concentrations higher than 300 Bq/m^3^ (Martín de Bernardo and Ruano, manuscript in preparation). This study is a continuation of preliminary work showing that a significant percentage of Spanish workers in radon-prone areas might be exposed to high radon concentrations, depending upon the construction material used in buildings and age of the individual building [45]. There are important differences depending on the type of work and also related to the building where the workplace is located. Older structures and those buildings made of granite tend to have higher radon concentrations, mainly due to a poorer isolation from the underlying rocks [76].

## Other effects of indoor radon

Currently, indoor radon has been only causally associated with lung cancer, and this is the message that should be communicated to general population. Nevertheless, there is ongoing research regarding its potential association with other diseases, oncologic or non-oncologic, and for some of them, evidence is accumulating regarding a possible role.

### Oncologic diseases other than lung cancer

Radon effect has been studied on oesophageal, stomach, brain, kidney, and other tumors [37, 39, 41, 42, 77, 78] but also in other non-solid tumors such as leukemia (both in adults and in children), lymphoma, and other uncommon tumors [79–82]. The evidence points to some association for some of them, mainly stomach and brain tumors [39, 41, 77] and also for leukemia [80]. Interestingly, the existence of an association is evidenced mostly in those areas previously classified as radon-prone, since it is easier to analyze whether a dose–response pattern exists, which is a well-known causation criterion. Many of these studies have been performed in Galicia.

### Non-oncologic diseases

Radon exposure has been studied as a possible cause of Alzheimer’s dementia and other neurologic diseases, and is also being studied as a contributing factor for inflammatory bowel disease (mainly ulcerative colitis and Crohn’s disease [47, 51, 83]). The evidence is still sparse and more research is needed. The most intriguing disease for which data on radon exposure as a contributing factor are accumulating is Chronic Obstructive Pulmonary Disease (COPD). Research in the framework of the Cancer Prevention Study II showed an association between COPD mortality and radon exposure [84]. An important limitation is that radon concentrations were estimated at the county level, but no individual measurements of participants were available. Two spanish studies have been published addressing the possible association of radon exposure with COPD. The first included 189 cases and 747 controls and observed that, while radon seemed not to be associated with COPD onset, radon exposure might increase the risk posed by tobacco consumption, acting as an effect modifier [50]. A further study (exclusively in never-smokers) is ongoing. This study, funded by the Instituto de Salud Carlos III has a multicentric, case–control, hospital-based design and has included close to 300 never-smoking COPD cases and 425 controls at this time. The preliminary results show that radon concentration is higher in COPD cases compared to controls (manuscript in preparation). Finally, a systematic review on this association concludes that this link between radon and COPD might be present and warrants more research [48].

## Radon, lung cancer, and other diseases: a look to the future

Radon is a known carcinogen with a well-established causal association with lung cancer. No additional research is needed on the association of indoor radon exposure with this disease or its histological types. However, the evidence on the potential molecular pathways that makes radon a carcinogen is sparse, despite pioneering research published in 2016 by Ruano-Ravina et al. on EGFR and ALK genes suggesting association in never-smokers [38]. These pathways may involve susceptibility genes, driver genes, tumor suppressor genes, and many others. While GWAS studies have been performed on small cell lung cancer including radon exposure [85], more research is clearly needed in this regard. To advance our understanding of the molecular pathways related to radon exposure and lung cancer onset, the Spanish Lung Cancer Group has funded the Radon-Atlas study, which aims to measure indoor radon in 500 Spanish lung cancer cases and combine these results with NGS sequencing. These data will provide important information on which genetic alterations are potentially associated with the highest radon concentrations. At this time, more than 150 patients have been included from more than 10 Spanish hospitals and we expect that this research will provide important answers on precisely how, at the molecular level, radon causes lung cancer.

There are also relevant questions on the potential association of radon exposure with the induction of other diseases, both oncologic and non-oncologic, albeit mainly respiratory diseases. This new research requires important epidemiological input; it is crucial to actually assess exposures and to place radon devices at all participants’ homes (instead of estimating indoor radon concentrations). There is a high likelihood that radon exposure will be associated with the genesis of other diseases, particularly with tumors, in the coming years due to the mounting evidence that is being accumulated.
